# Cellular Signaling and Production of Galactose-Deficient IgA1 in IgA Nephropathy, an Autoimmune Disease

**DOI:** 10.1155/2014/197548

**Published:** 2014-07-23

**Authors:** Colin Reily, Hiroyuki Ueda, Zhi-Qiang Huang, Jiri Mestecky, Bruce A. Julian, Christopher D. Willey, Jan Novak

**Affiliations:** ^1^Department of Microbiology, University of Alabama at Birmingham, 845 19th Street South, BBRB 762, Birmingham, AL 35294, USA; ^2^Department of Medicine, University of Alabama at Birmingham, 845 19th Street South, Birmingham, AL 35294, USA; ^3^Department of Radiation Oncology, University of Alabama at Birmingham, 845 19th Street South, Birmingham, AL 35294, USA

## Abstract

Immunoglobulin A (IgA) nephropathy (IgAN), the leading cause of primary glomerulonephritis, is characterized by IgA1-containing immunodeposits in the glomeruli. IgAN is a chronic disease, with up to 40% of patients progressing to end-stage renal disease, with no disease-specific treatment. Multiple studies of the origin of the glomerular immunodeposits have linked elevated circulating levels of aberrantly glycosylated IgA1 (galactose-deficient in some *O*-glycans; Gd-IgA1) with formation of nephritogenic Gd-IgA1-containing immune complexes. Gd-IgA1 is recognized as an autoantigen in susceptible individuals by anti-glycan autoantibodies, resulting in immune complexes that may ultimately deposit in the kidney and induce glomerular injury. Genetic studies have revealed that an elevated level of Gd-IgA1 in the circulation of IgAN patients is a hereditable trait. Moreover, recent genome-wide association studies have identified several immunity-related loci that associated with IgAN. Production of Gd-IgA1 by IgA1-secreting cells of IgAN patients has been attributed to abnormal expression and activity of several key glycosyltransferases. Substantial evidence is emerging that abnormal signaling in IgA1-producing cells is related to the production of Gd-IgA1. As Gd-IgA1 is the key autoantigen in IgAN, understanding the genetic, biochemical, and environmental aspects of the abnormal signaling in IgA1-producing cells will provide insight into possible targets for future disease-specific therapy.

## 1. Introduction

IgA nephropathy (IgAN) is the most common primary glomerulonephritis. IgAN was first described by Berger and Hinglais in 1968 based on observation of “intercapillary deposits of IgA-IgG” in renal tissue of patients with hematuria [1]. The initial manifestation of disease is frequently synpharyngitic hematuria, that is, macroscopic hematuria concurrent with upper respiratory tract infection [1, 2]. Progressive renal damage often culminates in end-stage renal disease (ESRD) that requires dialysis or transplantation as renal replacement therapy [3, 4]. In patients undergoing renal transplantation, the high rate of recurrence, up to 50–60%, suggests that the disease is of extrarenal origin [5]. Diagnosis of IgAN requires a renal biopsy and is based on detection of IgA immunodeposits in the glomerular mesangium [2]. Glomerular pathological changes may include mesangial hypercellularity and matrix expansion, variable degrees of segmental scarring, and crescent formation [6–8]. Fibrotic changes in the interstitium are observed in patients with more severe disease.

Human IgA has two subclasses, IgA1 and IgA2, with IgA1 constituting about 85% of the total IgA in the circulation. IgA in the glomerular deposits of IgAN patients is exclusively of the IgA1 subclass [9] and predominantly in polymeric form. IgA1 is produced in two distinct molecular forms, monomeric and polymeric (the latter consisting of 2–4 J-chain-linked monomers) [10]. IgA1-producing cells in mucosal tissues secrete predominantly polymeric IgA1; in contrast, the monomeric form accounts for >90% of the IgA1 in the circulation [10].

**Figure 1:**
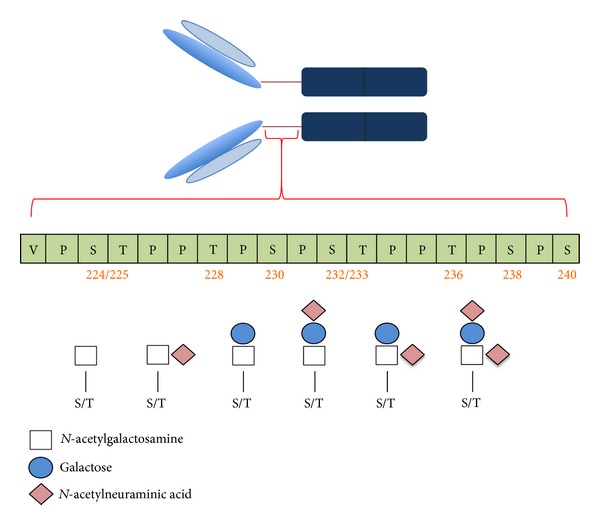
IgA1 and hinge-region* O*-glycosylation in IgAN patients. Figure of monomeric IgA1, with the hinge-region amino-acid sequence below to show the S/T residues that are* O*-glycosylated (numbered in red). Three to six core 1* O*-glycans are attached per hinge region, with* N*-acetylgalactosamine (white square) being the first sugar added; galactose (blue circle) may be then attached. Both glycans may be sialylated by* N*-acetylneuraminic acid (pink diamond), attached to* N*-acetylgalactosamine in an *α*2,6 linkage and to galactose in an *α*2,3 linkage. Patients with IgAN have elevated levels of circulatory IgA1 with some galactose-deficient* O*-glycans, consisting of terminal or sialylated* N*-acetylgalactosamine.

IgA1 contains both* N*-and* O-*linked glycans. In* N-*glycans, the first sugar added,* N*-acetylglucosamine (GlcNAc), is linked to a nitrogen atom in a N-X-S/T sequence motif, whereas* O-*glycans have* N*-acetylgalactosamine (GalNAc) bound to the oxygen atom of serine or threonine. Both subclasses of IgA are* N-*glycosylated, but only the IgA1 subclass has* O-*glycans. IgA1* O*-glycosylation sites are located in the hinge region, with 3–6* O*-glycans per heavy chain. IgA1* O*-glycans are core 1 structures that normally consist of GalNAc with a *β*1,3-linked galactose (Gal). The final extension of the core 1 structure is an addition of sialic acid to Gal, GalNAc, or both (Figure 1) [11–13].

**Figure 2:**
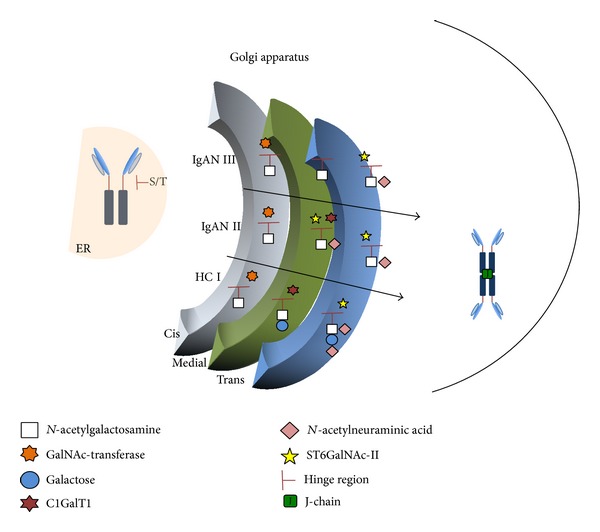
Pathways of IgA1* O*-glycosylation. The hinge region of IgA1 is* O*-glycosylated in the Golgi apparatus, a structure composed of multiple layers compacted together, with three designated regions: cis, medial, and trans. These structures support differential localization of various glycosyltransferases, an arrangement that confers a level of specificity and order of addition of sugar moieties.* Path I* shows normal (in healthy controls; HC) sequential addition of* N*-acetylgalactosamine (GalNAc), galactose (Gal), and* N*-acetylneuraminic acid (NeuAc) by their respective enzymes, GalNAc-transferase, core 1 galactosyltransferase (C1GalT1), and sialyltransferases, ST6GalNAc-II (adds NeuAc to GalNAc) and/or ST3Gal (adds NeuAc to Gal). There are multiple GalNAc-transferases that can initiate IgA1* O*-glycosylation.* Path II* shows a possible deviation in the localization of ST6GalNAc-II that could lead to a galactose deficiency due to premature sialylation of GalNAc. The addition of sialic acid blocks a later addition of galactose.* Path III* shows a scenario in which a reduced expression of C1GalT1 in IgA1-secreting cells from IgAN patients would decrease the addition of galactose to IgA1.* Pathways II and III represent possible deviations from normal glycosylation that could lead to production of galactose-deficient IgA1.* The polymeric form of IgA1 is formed after exit of IgA1 from the Golgi apparatus through the addition of J chain that binds covalently to the tail pieces of the heavy chains. The resultant polymeric IgA1 may be a dimer or higher oligomer.

Patients with IgAN have elevated serum levels of IgA1 that lacks Gal in one or more* O*-glycans, thus presenting with a terminal GalNAc or sialylated GalNAc (Gal-deficient IgA1; Gd-IgA1) [14]. Upon completion of glycosylation, IgA1 moves out of the Golgi apparatus and the monomers are processed into polymeric forms by addition of joining the (J) chain at the tail end of the heavy chain (Figure 2) [10, 15, 16]. Currently, J chain is not thought to play a role in Gd-IgA1 production, as it does not bind to IgA1 until after* O*-glycosylation occurs and IgA1 exits the Golgi apparatus [17].

A major difference between IgA1 and IgA2 is the extended hinge region in IgA1 heavy chain with nine potential* O*-glycosylation sites, of which up to six are occupied. An early study reported decreased binding of IgA1 from sera of patients with IgAN to jacalin (a lectin specific for GalNAc-Gal disaccharide) compared to that in sera of healthy controls [18]. These observations were later explained by the discovery of elevated serum levels of Gd-IgA1 in IgAN patients [14, 19, 20]. This finding prompted the hypothesis that a lower degree of* O*-glycan galactosylation on this immunoglobulin plays a role in the pathogenesis of IgAN. Indeed, the glomerular deposits of IgA1 of IgAN patients are enriched with Gd-IgA1 [21, 22]. Gd-IgA1 in the blood is primarily in the polymeric form and bound in circulating immune complexes (CIC). Investigators interpreted this finding to indicate that either systemic IgA1-producing cells altered the molecular form of secreted IgA1 or that mucosa-based IgA1-secreting cells had migrated to a location more proximal to the systemic blood flow [23].

In most IgAN patients the serum Gd-IgA1 levels are elevated compared to those of the normal population. In multiplex families with two or more IgAN patients (familial IgAN), almost half of the first-degree relatives have elevated Gd-IgA1 levels, although most exhibit no urinary abnormality [24]. Similarly, for patients with apparently sporadic IgAN (no relative with known kidney disease), about a quarter of asymptomatic blood relatives have an elevated serum Gd-IgA1 level. In contrast, about 5% of the general population and married-in relatives of patients with IgAN have a high serum level of Gd-IgA1. These studies are consistent with a hereditary factor affecting serum Gd-IgA1 levels. Segregation analysis indicated a likely influence of a major dominant gene, on a polygenic background, on the serum Gd-IgA1 level [24]. As most blood relatives with a high serum Gd-IgA1 level are apparently unaffected, elevated amounts of Gd-IgA1 alone are not sufficient for development of clinically apparent IgAN [24].

Previous research has shown that normal metabolism of IgA1 can be slowed by terminal Gal deficiency. IgA1 with enzymatically removed terminal Gal exhibits a longer clearance interval after injection in mice [25]. In human as well as murine hepatocytes, IgA1 is taken up by the asialoglycoprotein receptor that recognizes terminal GalNAc, but not if it has undergone sialylation [26, 27]. It should also be considered that, if Gd-IgA1 is bound by anti-glycan antibody, the aggregate complex is too large to pass into the space of Disse to reach the hepatocytes [28]. Together, these studies indicate that increased serum Gd-IgA1 levels could be related to lower hepatic catabolism rather than simply an increased production.

Because elevated serum Gd-IgA1 levels are associated with IgAN but likely not sufficient to induce clinically manifested disease, postulated models for the mechanisms of disease evolved to a proposal that multiple hits are required for renal injury. Several hypotheses tried to explain CIC formation and renal deposition, including Gd-IgA1 “aggregation,” Gd-IgA1 forming complexes with CD89 (IgA Fc-specific receptor), formation of complexes with environmental or microbial antigens, circulating antibodies that recognized Gal-deficient moieties on Gd-IgA1, and Gd-IgA1 directly binding to extracellular matrix proteins in the glomerular mesangium due to a novel sugar motif [29–32]. It is now thought that circulating Gd-IgA1 is recognized by anti-glycan autoantibodies of the IgA1 and/or IgG isotype, resulting in formation of CIC [20, 33]. Some of these pathogenic (nephritogenic) CIC deposit in the glomerular mesangium and activate resident mesangial cells, thus initiating glomerular injury [9, 34].

Recent genome-wide association studies (GWAS) of Han Chinese, European, and American Caucasian populations found several loci that were associated with IgAN [35–38]. These loci comprise genes encoding multiple components of the immune system, including the complement pathway (complement factor H-related genes 1 and 3,* CFHR1,3*); adaptive immunity (major histocompatibility complex,* MHC*); innate immunity, growth factors, and cytokines (*LIF* and* OSM* in the* HORMAD2* locus; human *α*defensin*, DEFA*; and tumor necrosis factor superfamily 13,* TNFSF13*) (Table 1) [36, 37]. The distribution of risk alleles in the loci differed between ethnic groups. Specifically, East Asian populations contained the most risk alleles, followed by Europeans, then Africans with the fewest. The risk differences among the groups were also reflected in the US, such that prevalence rates of IgAN are highest among Asian populations, with progressively lower rates in Caucasians and African-Americans. When effect size of each allele was taken into account, the differences in risk association between these groups were further enhanced [39, 40].

Two of the IgAN-associated loci contain genes for cytokines that may affect the circulating levels of IgA1. One locus, the Chr. 22q12* HORMAD2* locus, encompasses two cytokines, leukemia inhibitory factor (LIF) and oncostatin M (OSM) (Table 1). The IgAN risk allele in this locus is associated with elevated serum levels of IgA [36]. LIF and OSM are IL-6-related cytokines that signal through gp130 coupled to their respective cytokine-specific receptor(s) [41]. This family of cytokines has received some attention in the IgAN field, as the level of IL-6 is increased in the circulation of some IgAN patients [42]. Notably, LIF and OSM modulate mucosal immune responses. Recent* in vitro* studies, using IgA1-producing cells from IgAN patients stimulated with IL-6 family of cytokines, have demonstrated that these cytokines enhanced Gd-IgA1 production in IgA1-secreting cells from IgAN patients [17]. The effects on glycosylation of IgA1 produced by IgA1-secreting cells from healthy controls were minimal, suggesting differential responses of the cells from IgAN patients. A second IgAN-associated locus encompasses the* TNFSF13* gene that encodes APRIL (a proliferation-inducing ligand), a cytokine involved in augmenting IgA production in B cells independently of T-cell activation (Table 1) [43]. In Han Chinese with IgAN, a risk allele in this locus is associated with higher levels of serum IgA [37]. The effect of IgAN risk alleles on the expression of the encoded proteins, such as APRIL, is not known, nor is the mechanism by which APRIL may affect production of Gd-IgA1 well understood. How signaling by growth factors and cytokines in IgA1-producing cells from IgAN patients differs from that in healthy individuals is still being investigated, but there is evidence for involvement of enhanced activation of the JAK/STAT pathway [44–46].

Currently, there is no disease-specific treatment of IgAN and up to 40% of patients progress to ESRD within 20 years of diagnosis by biopsy [47, 48]; new targets for future disease-specific treatments are needed [48]. A reduction in serum Gd-IgA1 levels would decrease formation of nephritogenic CIC leading to less renal injury. We hypothesize that targeting a specific signaling pathway(s) would normalize expression, activity, and/or localization of specific glycosyltransferase(s) and thereby reduce Gd-IgA1 production. Manipulation of some of the unique signaling pathways promoting production of Gd-IgA1 in IgAN may constitute targets for therapeutic intervention.

## 2. Gd-IgA1 in IgAN

The role of circulating Gd-IgA1 in the formation of immune complexes was not fully appreciated until IgA1-IgG CIC were isolated and characterized. The Gd-IgA1 was found to be bound to IgG directed against hinge-region* O*-glycans, without Gal [20, 32, 33, 49]. Subsequent studies revealed that serum levels of Gd-IgA1 and the anti-glycan antibodies were associated with progression to ESRD [50, 51]. Thus, Gd-IgA1 and the corresponding autoantibodies are important in the pathogenesis of IgAN.

A unique resource has been developed for studies of the mechanisms of aberrant* O*-glycosylation of IgA1: EBV-immortalized IgA1-producing cells derived from peripheral blood mononuclear cells of patients with IgAN and healthy and disease controls. Notably, the degree of Gal deficiency of IgA1 secreted by the cell lines mirrored that of IgA1 in serum of the donors [52]. Thus, EBV immortalization and cloning of IgA1-producing cells did not significantly change the* O*-glycosylation pattern of the secreted IgA1. Using IgA1-producing cell lines, we have identified changes in some signaling pathways, such as STAT3, and activities of key glycosylation enzymes for the* O*-glycosylation of IgA1 [45, 46, 52]. These cell lines therefore represent a unique resource for in-depth studies of the glycosylation of IgA1.

## 3. *O*-glycosylation Pathways and Glycosyltransferases Involved in Gd-IgA1 Production

*O*-glycosylation takes place primarily within the Golgi apparatus that consists of multiple vesicles compacted into cis, medial, and trans compartments. These compartments function to delineate specific posttranslational modifications that help to control the order of addition of sugars to proteins. Glycosyltransferase enzymes reside in specific compartments of the Golgi apparatus, allowing the cell to control the glycosylation process at multiple points through localization, expression, and activity of specific enzymes [53, 54].

The initiation of* O*-glycosylation on glycoproteins by the attachment of GalNAc to a serine or threonine is catalyzed by GalNAc-transferases (GalNAc-Ts) [55]. For IgA1, this process is thought to be mediated primarily by GalNAc-T2, but other GalNAc-Ts are abundantly expressed in IgA1-producing cells and may contribute as well [56]. Core 1 *β*1,3-galactosyltransferase (C1GalT1) adds Gal to GalNAc, generating a GalNAc-Gal disaccharide. Expression of a C1GalT1-specific chaperone (Cosmc) is necessary for stability of the nascent C1GalT1 protein. Sialyltransferases can then add sialic acid to either saccharide or both. In IgA1-producing cells, *α*-*N*-acetylgalactosaminide *α*-2,6-sialyltransferase 2 (ST6GalNAc-II) adds sialic acid to GalNAc and galactosyl-*α*-*N*-acetylgalactosaminide *α*-2,3-sialyltransferase (ST3Gal) adds sialic acid to Gal [57–60].

Analysis of the expression and activity of key enzymes in IgA1-producing cells revealed important differences between cells from patients with IgAN and those from healthy and disease controls, such as patients with other glomerular diseases (including membranous nephropathy, minimal change disease, or immune-complex-associated lupus nephritis) [52]. Specifically, expression of C1GalT1 and Cosmc was lower and, conversely, expression of ST6GalNAc-II was higher in the cells from IgAN patients versus controls. All core 1* O*-glycosylation structures have GalNAc as the first sugar in the series and, as depicted in Figure 2, tend to be localized within the cis region of the Golgi apparatus. C1GalT1 is expressed at lower levels in the cells from IgAN patients compared to controls. The decrease in C1GalT1 levels in IgAN cells may also result from the decreased levels of C1GalT1-specific chaperone, Cosmc [45, 61]. The attachment of Gal to GalNAc likely occurs in the medial section of the Golgi apparatus due to the predominant localization of C1GalT1 there (Figure 2, pathway I). ST6GalNAc-II is overexpressed in IgA1-producing cells from IgAN patients. As sialylation of GalNAc in the disaccharide occurs late in the glycosylation process, ST6GalNAc-II is likely compartmentalized in the trans section of the Golgi apparatus. Migration of this enzyme to locations more proximal than that of GalNAc-Ts could lead to premature sialylation of GalNAc, before the addition of Gal (Figure 2, pathway III) [56], that inhibits subsequent attachment of Gal [46]. Thus, changes in the expression and/or localization of key enzymes may explain the differences in the pattern of* O*-glycosylation of IgA1 between IgAN patients and controls.

## 4. Signaling in IgA1-Producing Cells

Even with a genetic predisposition to elevated serum Gd-IgA1 levels in patients with IgAN, the onset of the disease requires a trigger resulting in the production of pathogenic CIC containing Gd-IgA1. The molecular nature of the trigger(s) is currently unknown, but it is well recognized clinically that upper respiratory tract infections are frequently associated with the first apparent manifestation of disease, “synpharyngitic hematuria,” that, in some patients, may be recurrent [62, 63]. It has been shown that these episodes of disease activity are associated with elevated serum levels of Gd-IgA1-containing CIC [51, 64–66]. This clinical association suggests a mucosal origin of an altered immunity in IgAN. This assumption is supported by findings from GWAS. One of the GWAS IgAN-related susceptibility loci, chromosome 17p13, contains the* TNFSF13* gene encoding APRIL [36]. APRIL is involved in T-cell-independent generation of IgA-secreting plasma cells and IgA class switching [43]. Serum levels of APRIL are elevated in some patients with IgAN and the 17p23 risk variant is associated with elevated levels of serum IgA [67]. Moreover, overexpression of a related factor, B-cell activating factor of the TNF family (BAFF), results in autoimmune disease with commensal microbiotoa-dependent glomerular IgA deposits in mice [67]. Furthermore, another IgAN-associated locus, encompassing several genes including those encoding LIF and OSM, also influences serum IgA levels. Risk alleles in this locus are associated with elevated serum IgA levels [36]. LIF and OSM belong to the IL-6 family of cytokines and are expressed in mucosal tissues where they exert immunoregulatory effects [68]. Together, these data implicate growth factors APRIL and BAFF and IL-6 family cytokines in the pathogenesis of IgAN.

Based on the accumulated clinical observations and biochemical and genetic data, we hypothesize that the onset and/or severity and progression of the disease depend on the activation of the immune system and that cytokines and B-cell growth factors play important roles in that aspect. Namely, these factors regulate Gd-IgA1 production and B-cell and plasma-cell maintenance and proliferation. This hypothesis offers several possible scenarios: (1) Gd-IgA1-producing B-cell-specific proliferation and survival are enhanced during mucosal infections, (2) B-cell signaling is altered and directly regulates expression and activity of specific glycosyltransferases, and (3) there is a direct signaling effect on the function of the Golgi apparatus in IgA1-producing cells that increases synthesis of Gd-IgA1.

**Figure 3:**
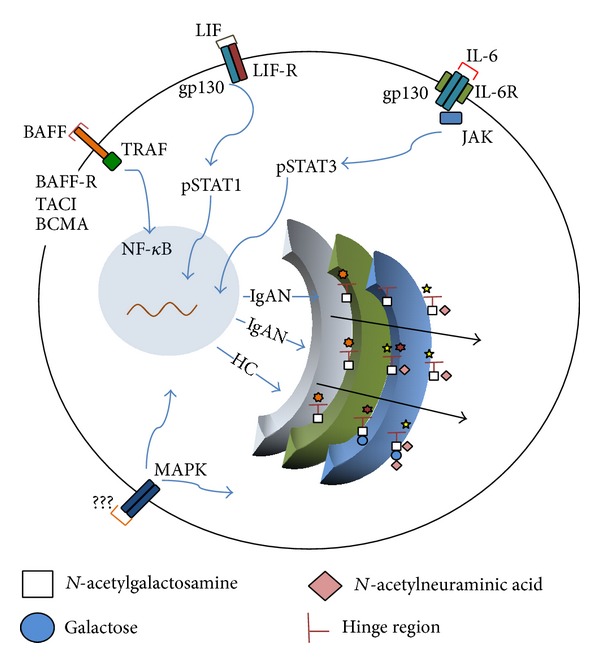
Examples of potential signaling mechanisms involved in the* O*-glycosylation of IgA1. Several signaling mechanisms likely regulate* O*-glycosylation patterns of IgA1 in the Golgi apparatus. In this scheme, we have highlighted examples of three types of signaling systems that are potential players in the production of galactose-deficient IgA1 (Gd-IgA1). BAFF signaling through the BAFF receptor(s) (TACI, BAFF-R, and BCMA/TRAF) prevents degradation of NF-*κ*B and enhances its subsequent translocation to the nucleus. This mechanism represents an example of a signaling pathway related to survival and proliferation of cells producing Gd-IgA1. Cytokine LIF, that binds the LIF-R/gp130 receptor complex, signals in B cells through STAT1 phosphorylation by JAK, followed by dimerization and translocation of the active STAT dimers to the nucleus. IL-6 activates the STAT3 pathway in B cells via JAK activation at the IL-6/gp130 complex. LIF and IL-6 signaling pathways are examples of a mechanism by which environmental factors may alter expression of specific glycosyltransferases. Activation of the MAPK pathways through various intraextracellular mechanisms can lead directly to the Golgi apparatus. MAPK signaling in B cells is another example of a pathway for an environment-mediated influence to change expression of specific glycosyltransferases, to signal directly to the Golgi apparatus, or both.

Currently, there is no robust animal model mimicking the pathogenetic pathways of human IgAN, mainly because the IgA1 isotype is present in only humans and hominoid primates. Serendipitously, an IgA-associated glomerulonephritis developed in mice overexpressing BAFF [67]. The manifestations included hematuria and albuminuria and were associated with IgA-containing CIC deposited in glomeruli [67]. BAFF, encoded by the* TNFSF13B* gene, is related to APRIL, sharing multiple receptors and functions. BAFF ligand can bind to several receptors, including BAFF-R (transmembrane activator), B-cell maturation antigen (BCMA), and the calcium modulator and cyclophilin ligand interactor (TACI), to activate the NF-*κ*B pathway (Figure 3). BAFF is a B-cell-survival cytokine and, in a mouse model of nephrotic syndrome, induces B-cell hyperplasia and higher serum levels of IgA. Notably, murine IgA resembles human IgA2 and lacks a hinge region for attachment of clustered* O*-glycans. Alterations in IgA production in humans could be due to class-switching recombination (CSR), changing from IgM or IgG to IgA upon stimulation with BAFF, which can affect B cells at mucosal interfaces [69, 70]. BAFF and/or APRIL levels are elevated in the circulation of some IgAN patients, suggesting a possible effect on circulatory B cells [67, 71]. Future studies will need to characterize mechanisms by which B-cell growth factors affect production of Gd-IgA1 in patients with IgAN and to answer many important questions. For example, do these factors affect IgA1* O*-glycosylation in IgA1-producing cells? Do these factors enhance survival of plasma cells already differentiated into Gd-IgA1-secreting cells that would not survive in healthy individuals? Is abnormal signaling in IgA1-producing cells from patients with IgAN induced by these growth factors?

Another class of proteins affecting IgA1 production includes IL-6 and related cytokines. IL-6 is a plasma-cell differentiation factor and canonical inducer of IgA1 production; its serum level is elevated in some IgAN patients [46]. IL-6 binds the IL-6R/gp130 complex and activates the JAK/STAT3 pathway, leading to nuclear translocation of STAT3 (Figure 3) [41, 68]. In IgA1-producing cells from IgAN patients, exposure to IL-6 prolongs and significantly increases STAT3 activation compared to cells from controls [72]. IL-6 signaling increases IgA1 production in IgA1-secreting cells from healthy controls and IgAN patients. Moreover, IL-6 enhances production of Gd-IgA1, but only in cells from IgAN patients, further supporting the role of immune activation in exacerbating the underlying pathology [73]. A possible mechanism for STAT3 to increase Gd-IgA1 production is through altered expression of specific glycosyltransferases: increased for ST6GalNAc-II and decreased for C1GalT1 [17, 46].

Activities of the corresponding enzymes mirror the changes in gene expression. These observations suggest a possible mechanism for IL-6/STAT3-mediated Gd-IgA1 production, one that involves regulation of expression and activity of key glycosyltransferases. Thus, changes in* O*-glycosylation patterns of IgA1 secreted by cells from IgAN patients reflect differences in signaling pathways that ultimately affect the Golgi apparatus.

Other IL-6-related cytokines, LIF and OSM, also activate B cells through similar receptors and associated STAT pathways [68]. The potential role of these cytokines in the pathogenesis of IgAN has been outlined in a GWAS report that found a significant correlation between SNPs in the* HORMAD2*  locus and prevalence of the renal disease. SNPs in this interval, rs2412971, were associated with decreased serum IgA1 levels in patients and span a region that contains* LIF* and* OSM* [40]. LIF and OSM cytokines have been tested on IgA1-producing cells from IgAN patients and controls; both increased production of Gd-IgA1, but only in the cells from IgAN patients [74].

The signaling mechanism involves binding of the ligands to the LIF receptor or OSM receptor that recruits gp130 and activates the JAK/STAT pathway (Figure 3). In IgA1-producing cells, STAT1 is phosphorylated and can induce Gd-IgA1 production in cells from IgAN patients, similar to the activity promoted by IL-6/STAT3-mediated signaling [41]. Notably, in most cases of JAK recruitment through IL-6 family members, downstream kinases are also phosphorylated, such as the mitogen-activated protein kinase (MAPK) pathway. MAPK pathways are known to modulate Golgi morphology and glycosylation functions, as outlined below.

A recent study by Chia et al. screened many signaling genes by using siRNA knockdown and assessed the individual effects on glycosylation patterns and morphology of the Golgi apparatus of HeLa cells [75]. Glycosylation was assessed for changes in terminal sugars using a panel of lectins that recognize specific glycans. These experiments showed that knockdown of dual-specificity phosphatases (DUSP), proteins that negatively regulate MAPK pathways, enhanced MAPK phosphorylation. DUSP siRNA knockdown altered the morphology of the Golgi apparatus and decreased Gal content of* O*-glycans on newly synthesized glycoproteins. Using this data set, it is possible to search for genes and pathways that may produce the same or similar* O*-glycosylation pattern as in the hinge region of Gd-IgA1 from patients with IgAN.

As signaling by IL-6/LIF/OSM family members affects IgA1 production in all cells but alters IgA1* O*-glycosylation only in cells from IgAN patients, we speculate that increased activation of a signaling pathway(s) plays a direct role in influencing the functions of the Golgi apparatus [46, 74]. Our preliminary data revealed that several signaling pathways, including MAPK, are enhanced in IgA1-producing cells from IgAN patients versus controls (unpublished data). Thus, it is conceivable that increased activation of the MAPK pathway, possibly through environmental factors, alters IgA1* O*-glycosylation through direct signaling into the Golgi apparatus in IgA1-producing cells of IgAN patients (Figure 3). These observations link signaling pathways with IgA1 aberrant glycosylation, a model that offers potential for testing new targets for manipulating* O*-glycosylation through downregulation of “inappropriate” signaling in IgAN.

## 5. Conclusions

Progressive deterioration of renal function in IgAN patients remains a significant concern, as current therapeutic options do not include a disease-specific approach. Treatment with ACEI/ARB to reduce proteinuria and control of hypertension and, for some patients, immunosuppressants can slow the loss of renal-clearance function [2, 76]. These therapeutic approaches are not tailored to IgAN. The ongoing renal injury would be better controlled with molecular therapies targeted for the disease. The current multihit hypothesis of the pathogenesis of IgAN, wherein Gd-IgA1 production in conjunction with anti-glycan antibodies creates CIC that drive the disease, suggests that limiting either of the two initial hits would provide a long-term benefit [77]. Recent studies have shown that several cytokine- and B-cell-specific survival/signaling pathways apparently play a role in driving aberrant glycosylation of IgA1. Further research is required to more clearly define how these pathways influence IgA1 glycosylation in B cells. Focusing on pathways to reduce production of Gd-IgA1 will likely provide opportunities for development of future disease-targeted treatment of patients with IgAN.

## Figures and Tables

**Table 1:** Susceptibility loci for IgAN from multiple GWAS [35–40].

SNPs associated with IgAN	Genes in the region
*MHC region *	
6p21: rs9275596 (T)	*HLA-DRB1, HLA-DQA1, HLA-DQB1 *
6p21: rs9275224 (A)	
6p21: rs2856717 (C)	
6p21: rs9357155 (G)	*PSMB8, PSMB9, TAP1, TAP2 *
6p21: rs1883414 (T)	*HLA-DPB1, HLA-DPB2, HLA-DPA1, *
	*COL11A2 *
*CFH and CFHR gene cluster *	
1q32: rs6677604 (G)	*CFH, CFHR *
*HORMAD2 locus *	
22q12: rs2412971 (A)	*HORMAD2, MTMR3, LIF, OSM *
*DEFA locus *	
8p23: rs2738048 (T)	*DEFA *
*TNFSF13 locus *	
17p13: rs3803800 (A)	*TNFSF13 (APRIL) *

GWAS: genome-wide association studies; MHC: major histocompatibility complex; SNP: single nucleotide polymorphisms; CFH: complement factor H; HORMAD2: HORMA domain containing 2; DEFA: human *α*-defensin; TNFSF13: tumor necrosis factor ligand superfamily member 13.
